# Improving the communication skills of medical students ——A survey of simulated patient-based learning in Chinese medical universities

**DOI:** 10.1186/s12909-022-03596-0

**Published:** 2022-07-13

**Authors:** Yurong Ge, Yuko Takeda, Peifeng Liang, Shilin Xia, Marcellus Nealy, Yoko Muranaka, Shishu Sun, Takao Okada

**Affiliations:** 1 Department of Teaching Affairs, The First Affiliated Hospital Northwest University for Nationalities, Yinchuan, China; 2 People’s Hospital of Ningxia Hui Autonomous Region, Yinchuan, China; 3 Department of Medical Education, Juntendo University, Tokyo, Japan; 4 Department of Medical Statistics, People’s Hospital of Ningxia Hui Autonomous Region, Yinchuan, China; 5 Clinical Laboratory of Integrative Medicine, the First Affiliated Hospital of Dalian Medical University, Dalian, China; 6 General Education, Juntendo University, Tokyo, Japan; 7 Graduate School of Health Care and Nursing, Juntendo University, Chiba, Japan

**Keywords:** Simulated patient, Medical education, Communication, Clinician -patient relationship

## Abstract

**Background:**

It is useful to advance simulated patient (SP) participation in teaching to improve the communication skills of medical students, so this study aims to explore the current state of Chinese mainland SP education.

**Methods:**

A cross sectional survey was designed utilizing well defined quantitative research methods and descriptive statistics. The questionnaire sought information which elucidated the current status of SP-based education, the origin of SP-based learning, SP training, challenges of this learning strategy and future developments. Questionnaires were distributed to 79 medical colleges in mainland China, and 68 were returned. Of these, 64 constituted valid responses (81%).

**Results:**

The number of SP-based education activities in medical colleges offering 5-year、7-year and 8-year clinical medicine programs was significantly higher than that in medical colleges which offered only a single 5-year program (*p* < 0.01). Communication skills training accounted for 73% of the content of SP-based learning activities, and was expected to rise in the future to 90%, in response to a need to improve doctor-patient relationships. Persons recruited as ‘simulated patients’ included students (21% of the total), residents (49%), medical staff (15%) and teaching staff (14%).

Colleges, planning a SP-based education program, preferred teachers (80%) and students (55%) to assume ‘simulated patient’ roles. In objective structured clinical education (OSCE) scenarios, co-scoring by both SPs and teachers featured more highly in the ‘consultation’ station and ‘doctor-patient communication’ station. A number of factors were identified as hindering future development and implementation of SP-based learning including budget restraints, SP selection and training.

**Conclusions:**

SP-based learning programs offer clear benefits for improving the clinical education of medical students and their communication skills. The main obstacles to achieving more widespread and higher quality SP-based education are insufficient funding and the lack of standardized training and performance evaluation processes for simulated patients. Medical colleges should consider reducing the proportion of students and teachers acting as SPs, and attract more citizens to participate in SP-based learning activities. Formalised training and evaluation of SPs performance are necessary to establish a ‘standard simulated patient’ for a particular medical discipline, thus improving SP-based activities and student learning.

**Supplementary Information:**

The online version contains supplementary material available at 10.1186/s12909-022-03596-0.

## Background

Simulated patient (SP)-based educational practices have been used since the 1960s. Simulated patients include both healthy individuals and patients who can accurately represent actual clinical problems after standardized and systematic training. Continuous development and improvement of SP-based education has resulted in this discipline being regarded, as an indispensable teaching resource particularly in an era of “patient-centered” healthcare [1, 2].

Simulated patient-based learning was introduced in China in 1991 [3] and is now widely and actively used in medical teaching institutions and hospitals throughout the country. SP-based learning is integrated the teaching programmes of various clinical disciplines, including internal medicine, psychiatry, traditional Chinese medicine, and nursing [3–7]. In 2003, Chan et al. [8] reported that significant improvement in communication skills among Chinese clinicians could be achieved through training that involved role-play and feedback using SP-based learning. Shen [4] reported that SP-based clinical teaching of internal medicine can significantly improve student performance and communication skills.

In recent years, the relationship between clinicians and patients in China has often become more difficult, leading to abuse and violence against medical staff in some cases. The reasons for this behavior are many and complex, but the communication barrier is considered to be one of them [9]. In response to this trend, a survey was conducted of SP-based education programs in medical universities across China to assess the current status of SP-based learning programs and their potential for improving doctor-patient communication.

## Methods

### Data collection

Following initial email contact and phone confirmation, we sent the online survey questionnaire to 79 medical colleges in mainland China, requesting that it be completed by the head of SP-based teaching. The survey was conducted for a period of one month in October 2019, with reminder emails sent 2 and 3 weeks after the initial request. With regard to consent to participate, an additional file shows this in more detail [see Additional file 1], the purpose of the survey was stated in an email, which was directly sent to the participants. The participants chose whether or not to respond without any penalty. In addition, they were informed that filling out of the survey was considered to be the same as giving consent. Documentation of informed consent was secured at the beginning of the survey, and all participants have read and agreed to the informed consent agreement. The informed consent was obtained from all subjects. Eleven colleges declined to participate due to the lack of SP-based programs at the institutions. We received 68 completed questionnaires(response rate 86%). Four questionnaires were excluded from the analysis due to missing data.

Medical colleges in China offer either a single 5-year clinical program, or long programs of 5, 7 or 8-year duration. Medical students graduate with a bachelor’s degree after 5-years study. The 7-year program involves undergraduate and masters study, and a master's degree is obtained after graduation. The 8-year program combines undergraduate, masters and doctoral study, and a doctoral degree is obtained on graduation.

### Questionnaire design

The first draft of the questionnaire was based on the SP series of questionnaires developed by Abe [10]. Yurong Ge oversaw the development of the questionnaire. It was approved by 3 Japanese medical education experts, 3 Chinese medical education experts, and 1 statistician through the Delphi expert method (Delphi Expert Consultation) [11] to demonstrate and revise. Opinions were collected and the questionnaire further revised through a pre-survey involving 10 SP-trained teachers at Northwest University for Nationalities and Ningxia Medical University.

The questionnaire comprised 37 items; the main elements being the current status of SP-based education, the origin, SP training course, challenges and future plans. The questionnaire consisted of binary response items (YES/NO), multiple-choice questions and subjective response.

### Statistical analysis

The data collected from the survey was statistically processed using SPSS 20 (SPSS, Inc., Chicago, IL, USA). Quantitative data was expressed as frequency and percent. Chi-square test or Fisher’s Exact Test were used for 2 × 2 quantitative variables and *p* < 0.05 was considered to indicate a statistically significant difference.

## Results

### Test for the questionnaire

The value of the Kronbach coefficient that I have obtained from the reliability test of the self-designed questionnaire was 0.76, indicating that it demonstrated good reliability.

### SP characteristics in medical education

Of the 64 colleges included in the data analysis, 37 (58%) incorporated SP-based learning in the degree program. Forty four (69%) medical schools offering only a 5-year medical degree, completed the survey. Of these, less than half (20 schools, 46%) carried out SP-learning activities. Table 1 shows that a significantly greater proportion of schools offering the longer seven-year and eight-year degrees included SP-based learning in the programme (85%, 17 of 20 respondents) (*p* < 0.01). The history of providing SP training in single 5-year clinical program colleges and long programme colleges is shown in Table 2. More colleges offering long program training had instituted SP education for more than a decade (*p* < 0.001).

**Table 1:** The quantity of SP medical colleges with or without SP program (*n* = 64)

Length of Program	Program involved SP [n(%)]	Program not involved SP [n(%)]
5 years	20 (45.5)	24 (54.5)
5–7 years (or 8 years)	17 (85.0)	3 (15.0)

χ^2^=8.816, *p < 0.01

*SP* Simulated patients

**Table 2:** Time length of SP program implementation (*n* = 37)

Length of Program	Less than 10 years [n(%)]	Less than 10 years [n(%)] 10 years or above [n(%)]
5 years	19 (95.0)	1 (0.5)
5–7 years (or 8 years)	19 (95.0)	12 (70.6)

Fisher’s Exact Test: p* < *0.001*

Twelve of the 20 colleges providing the longer 7- and 8-year medical degree programs had instituted SP-based education for more than a decade. Four could point to a history of using SP-based learning of 15 years or longer. All universities which had inculcated SP-based learning activities in the medical degree for more than 10 years had been established for more than 60 years.

**Fig. 1:**
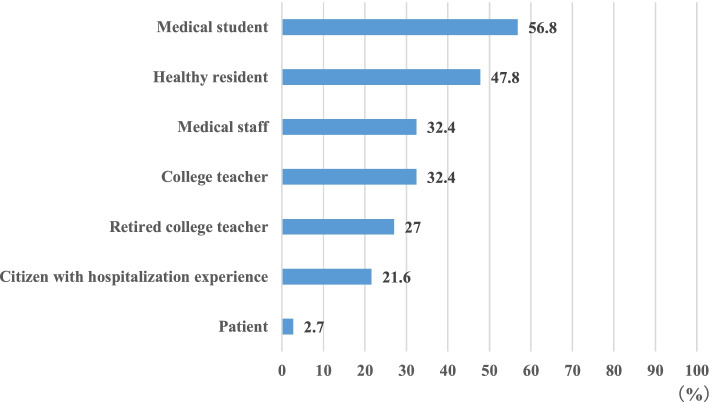
Types of SP member (*n* = 37)

At the time of survey, a total of 1004 persons were involved in playing SP roles in the responding schools, of which, 438 were male (44%) and 566 were female (56%). Figure 1 shows that colleges employed more than one type of person as SPs; 21 out of 37 colleges (57%) recruited medical students, while local residents were recruited at 18 colleges (49%), Medical staff and teachers played the role of SP at 32% of medical schools.

**Fig. 2:**
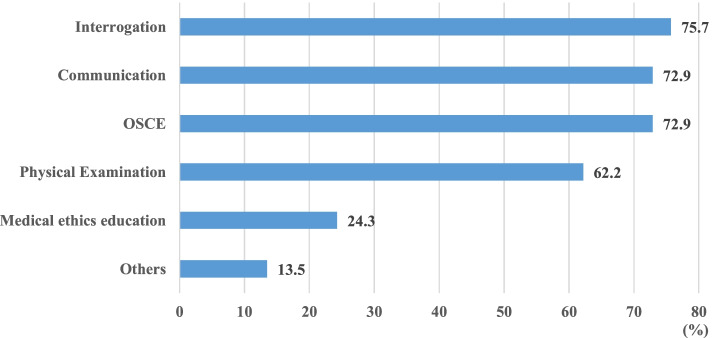
Types of SP Programmes (*n* = 37). OSCE: Objective Structured Clinical Examination. SP: simulated patients

**Fig. 3:**
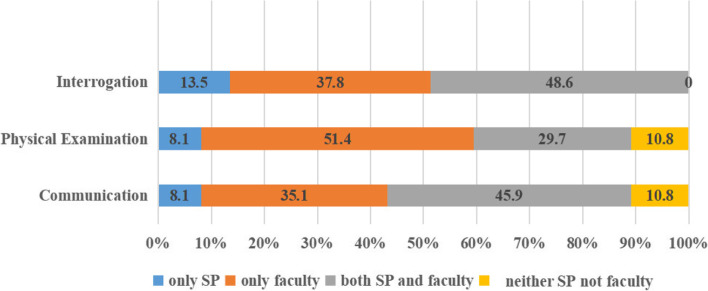
Categories of OSCE Evaluator (*n* = 37). OSCE: Objective Structured Clinical Examination

The main types of SP-based learning activity are shown in Fig. 2, namely consultation (78.4%), objective structured clinical evaluation (OSCE) (75.7%), and doctor-patient intercommunication (72.9%). During the OSCE activity (Fig. 3), SPs and examiners gave a co-score for students' performance in consultation (48.6%), doctor-patient communication (45.9%), and physical examination (29.7%). Only a small number of colleges(10.8%) gave SP’s score without a co-examiner evaluation.

### SP training and evaluation

Of the 37 institutions that featured SP-based learning activities 28 (76%) had a department responsible for SP training, which was delivered mostly through lectures but also included field observation, demonstrations, videos, and practice exercises. All 37 universities formally assessed SP performance, by means of feedback from faculty (73.0%), students (70.3%) and the use of a self-designed scale (29.7%). Universities without quality evaluation of SP performance accounted for 16.2% of the 37. Thirty-one of the 37 universities (84%) responded that they were satisfied with the performance of current SPs.

### Challenges and future plans in SP-based education

**Fig. 4:**
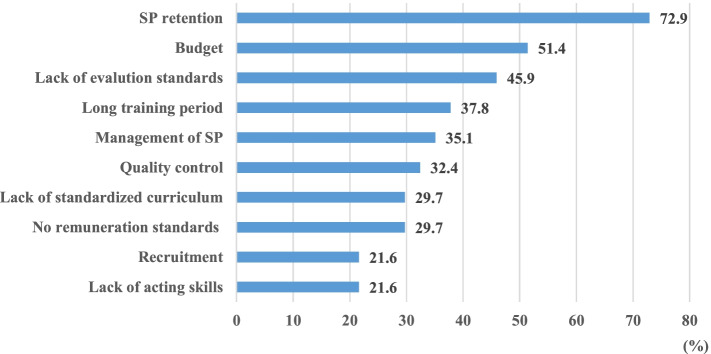
Challenging issues in implementing SP based program (*n* = 37). SP: simulated patients

The most challenging issues facing SP-based education were cited as “retention” (73%) followed by “insufficient budget” (51%) and “lack of evaluation standards” (46%) (Fig. 4). Responses also revealed that there were few standardized training courses in SP role playing.

**Fig. 5:**
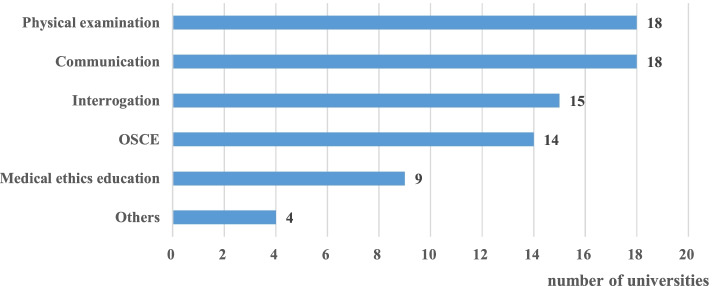
Topics for Future Implementation (*n* = 20). SP: simulated patients. OSCE: Objective Structured Clinical Examination

Twenty colleges (74%) indicated a desire to establish SP-based learning courses in the future specifically designed to augment “Communication” and “Physical examination” training (Fig. 5), and these universities are what we are focusing on. Eighteen colleges (90%) preferred to set up SP–based learning activities in doctor-patient communication and physical examination (Fig. 5). When asked about prerequisites for establishing SP programs at their respective universities, 18 (90%). colleges indicated a need for increased budget and SP trainers. When asked who would be considered as a SP, the most common response was faculty (70.37%), followed by general public (62.96%) medical personnel (30.07%), or students (55%, respectively).

Among the 27 colleges which did not include SP-based education in the medical program, 7 (25.9%) were not planning to utilize SP in the future. The reasons given included the set curriculum and lack of budget support, trainers, SP recruitment strategies and expert cooperation.

## Discussion

This study was designed to investigate the current state of development of SP-based learning in Chinese Medical Universities. The survey response rate exceeded 80% and revealed that almost 60% of responders had inculcated SP-based learning activities into the education programme. These findings are in line with the earlier study of Yang et al. [12] in 2016 who reported SP-based teaching activities in 60% of the 80 medical institutions surveyed. Our study also revealed that SP-based learning activities are mainly concentrated in consultation, physical examination, doctor-patient communication. Although SP-based learning activities have been widely used in China since 1991 and medical educators have expressed a high degree of satisfaction with the approach and its benefits for student education, the current survey identified major challenges facing the future provision of SP-based learning. Most colleges, planned to recruit medical student and teachers as SP role players. Although using students and faculty may be cost-effective, this practice may reduce the realism of training and thus limit its effectiveness. Training in doctor-patient intercommunication and ‘listening to patient’ skills has relied heavily on the participation of SPs who can contribute through their own experiences as patients [12, 13]. It is difficult to match this ‘real life’ experience using students and faculty. Furthermore, medical students are resident in college for a limited time, so that their useful period as SPs is short. Replacements need to be trained every year, which takes time and money [14].

An alternative is to utilize non-academics as SPs. The ‘patient-centered’ medical philosophy proposed by the World Health Organization (WHO), strongly advocates the participation of patients and the public in medical care, education and research [15]. Therefore, it is suggested that medical colleges should reduce the proportion of students and teachers acting as SPs and attract more citizens to participate in SP-based learning activities.

In recent years, the relationship between clinicians and patients in many countries including China, Japan, India, Pakistan and Nepal has often deteriorated, leading to abuse and violence against medical staff in some cases [16]. The reasons for this behavior are many and complex, but the communication barrier is considered to be an important factor [9]. A report of medical education in four European countries showed that communication skills improved when SP-based learning activities featured in the program [17]. In Japan SP-based activities in medical education, were mainly focused on doctor-patient communication [13]. The present survey revealed that SP-based communication skills training in Chinese medical colleges accounted for high proportion of SP-based activities and was expected to increase in colleges planning to include SP-based education. Thus, it is evident that Chinese medical colleges are seeking to improve doctor-patient relationships, with the required urgency, through enhanced communication skills.

The present survey highlighted a number of obstacles which are hindering future development and implementation of SP-based learning. In OSCE activities, almost 50% of responding colleges conducted student assessments based on input from both examiner and SP. The examiner’s score reflects professional clinical judgement, while, the SP’s score appears to reflect the student’s performance in CST through patient experience. Previous research has shown a positive association between examiner’s and SP’s score in OSCE activities [18]. Student CST has been shown to benefit greatly from SP feedback [15]. However, the quality of SP feedback depends on the quality of SP training [12]. A formalized system of training and evaluation of SPs and evaluation criteria are necessary to establish a ‘standard simulated patient’ for a particular medical discipline. In this way, training courses for SPs may be well defined, permitting objective grading of SP performance. rather than subjective feedback from student or teacher. Standard training courses for SPs already exist in Japan [19], but not yet in China and this shortcoming is recommended to be addressed. The same message appeared in the study of Yang et al. [12].

Currently, SP based education needs extra financial support, reassessment of SP recruitment principles, formalized training of SPs and establishment of criteria for assessing their performance. Investment in these areas is expected to improve the performance and stability of the SP team, the effectiveness of SP–based clinical education and doctor-patient relationship through enhanced communication skills.

## Conclusion

In line with world views, SP-based learning activities in Chinese medical colleges are generally recognized as contributing significantly to the education of medical students. The survey revealed that SP-based CST accounted for a high proportion of SP-based activities and was expected to increase in response to a need to improve doctor-patient relationships. A number of factors were identified as hindering future development and implementation of SP-based learning including budget restraints, SP selection and training. It is recommended that medical colleges consider reducing the proportion of students and teachers acting as SPs and attract more citizens to participate in SP-based learning activities. Formal training and evaluation of SP performance are necessary to establish a ‘standard simulated patient’ for a particular medical discipline, thus improving SP-based activities, student learning and doctor-patient relationships through enhanced communication skills. 

## Supplementary Information

**Additional file 1.**

## Data Availability

The datasets used and/or analyzed in the current study are available from the corresponding author on reasonable request.
