# Supplementary material for: Improving the communication skills of medical students ——A survey of simulated patient-based learning in Chinese medical universities
Source: BMC Med Educ. 2022 Jul 13;22:539. doi: 10.1186/s12909-022-03596-0 (PMC9281092; doi:10.1186/s12909-022-03596-0)
Supplement: Supplementary file 1 — Additional file 1. [file 12909_2022_3596_MOESM1_ESM.zip › informed consent.pdf]

Investigate and study informed consent

Dear teacher,

I am a visiting scholar in the Medical Education Research Office of Juntendo University in Japan, engaged in related research on Chinese medical education. This research topic is "National Survey on the Application Status of Standardized Patients in Medical Education in China", mainly to understand the training and application status of standardized patients in the teaching of medical school of your university. The survey results will be used for academic research, and your cooperation will provide scientifically based suggestions for medical education in China.

Note: 1. If you do not know about standardized patients in your school, please forward the email to the person in charge.

2. If you agree to cooperate, please reply to this email "agree" directly, and then we will send you the network questionnaire. If you have any questions, please feel free to contact the following personnel.

The following is a description of the research topic, looking forward to your understanding and cooperation.

1. Research object

From an international perspective, this study investigated the application status of standardized patients in medical colleges in China, in order to provide a scientific basis for medical education in China and promote the development of medical education.

2. The research methods

We sent informed consent to the university's standardized patient department (or head), after obtaining the consent, we will conduct the network questionnaire survey.

3. Research ethics

(1) All survey email addresses were obtained from the Internet.

(2) Because it is an online survey, your personal email information will not be displayed after submitting the questionnaire and the conclusion of the survey will be declared. Academic publications will not involve your university if they are not intended for purposes other than research.

(3) Because the questionnaire is submitted in a form that does not involve personal information, the questionnaire cannot be withdrawn after submission.

I confirm that the person have read this manuscript and given their permission for
it to be published in PEC.

Researcher: Yurong Ge and Peifeng Liang

Affiliation: Yurong Ge Teaching Management, People's Hospital of Ningxia Hui Autonomous Region; Visiting scholar, Medical Education Laboratory, Juntendo University, Japan

Peifeng Liang People's Hospital of Ningxia Hui Autonomous Region

Company address: Teaching Management Department, 4th Floor, Training Center Building, No.301, Zhengyuan North Street, Jinfeng District, Yinchuan, Ningxia

Website link: http://www.nxrmyy.com/

Contact: Peifeng Liang, 0951-5920194 or 13895085519

Email: Yurong Ge, 825829353@qq.com

Peifeng Liang, doctor_pf@126.com
Looking forward to your cooperation!
